# Multiple-level thresholding for breast mass detection

**DOI:** 10.1016/j.jksuci.2022.11.006

**Published:** 2023-01

**Authors:** Xiang Yu, Shui-Hua Wang, Yu-Dong Zhang

**Affiliations:** School of Computing and Mathematical Sciences, University of Leicester, Leicester LEI 7RH, United Kingdom

**Keywords:** Mass detection, Multiple-level thresholding, Deep CNNs

## Abstract

Detection of breast mass plays a very important role in making the diagnosis of breast cancer. For faster detection of breast cancer caused by breast mass, we developed a novel and efficient patch-based breast mass detection system for mammography images. The proposed framework is comprised of three modules, including pre-processing, multiple-level breast tissue segmentation, and final breast mass detection. An improved Deeplabv3+ model for pectoral muscle removal is deployed in pre-processing. We then proposed a multiple-level thresholding segmentation method to segment breast mass and obtained the connected components (ConCs), where the corresponding image patch to each ConC is extracted for mass detection. In the final detection stage, each image patch is classified into breast mass and breast tissue background by trained deep learning models. The patches that are classified as breast mass are then taken as the candidates for breast mass. To reduce the false positive rate in the detection results, we applied the non-maximum suppression algorithm to combine the overlapped detection results. Once an image patch is considered a breast mass, the accurate detection result can then be retrieved from the corresponding ConC in the segmented images. Moreover, a coarse segmentation result can be simultaneously retrieved after detection. Compared to the state-of-the-art methods, the proposed method achieved comparable performance. On CBIS-DDSM, the proposed method achieved a detection sensitivity of 0.87 at 2.86 FPI (False Positive rate per Image), while the sensitivity reached 0.96 on INbreast with an FPI of only 1.29.

## 1 Introduction

Medical image analysis has played a key role in the modern health system. By deploying early screening, cancers or diseases can be detected at an early stage and morbidity and death rates can be reduced (Siegel et al., 2021). For breast cancer, severity analysis can be achieved through an invasive method such as a biopsy. However, the patients have to go through a series of painful tissue extraction procedures and wait for a rather long time until the analysis is completed. To facilitate the diagnosis of disease pain-free in a more efficient way, experts in computer science have developed useful Computer-aided detection (CAD) systems. Those systems can be divided into traditional and deep learning-based ones. Traditional CAD systems usually consist of modules, including pre-processing, segmentation, feature extraction(and feature selection if needed), and classification. However, human intervention is usually an indispensable part of ensuring the success of these systems. Instead, deep learning-based systems can greatly minimize manual intervention as deep learning has brought great benefit to the development of many key areas such as autonomous driving, cyber-security, and medical imaging (Grigorescu et al.,2020; Berman et al., 2019; Xiang et al., 2021; Yolcu et al., 2020; Oztel et al., 2018). Compared to traditional CAD systems, deep learning-based CAD systems turn out to be more advantageous in terms of performance and robustness. Also, the individual modules, including feature extraction, selection and classification in conventional CAD systems, can be integrated into single deep learning architecture, which indirectly boosts the robustness of deep learning-based systems. However, some shortcomings still remain for deep learning-based CAD systems. One is the improvable robustness of these systems. While the performance of deep learning-based CAD systems on limited datasets can be promising, these systems, however, can still perform surprisingly badly on images collected by different imaging devices or the same devices with different settings. Another problem mainly comes from the size of available resources, including datasets and computing devices. The final performance of deep learning-based CAD systems is greatly determined by the size of available datasets and annotations. While there are numerous attempts to mitigate the situation (Bria et al., 2020; Taghanaki et al., 2021), the original size of the dataset remains a dominating factor impacting the performance of deep learning-based CAD systems. Especially annotating a dataset is an expensive procedure that requires a large sum of money for manual expenses and a large amount of time due to the challenges of providing accurate annotations. Also, as it was widely known, the training of large-scale deep learning models requires large computing resources such as GPUs. Therefore, the available computing resources and deployment of these models are also potential factors that hinder the spread of deep learning-based CAD systems.

Breast cancer, a common top-ranking cancer like lung, and prostate cancer, has been recognized as one of the major threats to women's health. While the coincidence rate of breast cancer increases, the death rate declines thanks to early screening procedures (Siegel et al., 2021). Considering factors including cost and efficiency, radiologists in the community recommend X-ray mammography as the key tool for the early detection of breast abnormalities. Given that manual interpretation of mammography is a time-consuming and challenging task, numerous CAD systems for mammography images have been developed to aid radiologists in the community during the past decades. Compared to other breast abnormalities such as calcification, distortion, and breast mass are the most significant symptoms of breast cancer. However, the intrinsic complicated nature and varied shapes of breast mass make it a challenging task to detect and segment breast mass. Additionally, the low signal-to-ratio of mammography images indirectly impairs the performance of hand-crafted feature-based CAD systems. Another challenge for successful breast mass detection is the varied density of breast tissues. When dense breast tissue is present, the pixel intensities of these tissues are close to real breast mass and may also overlap the breast mass. Therefore, it is usually more challenging to recognize and partition real breast masses breast tissue.

In this paper, we developed a novel breast mass detection system for mammography images with varied breast densities. A side benefit brought by the detection system is that coarse segmentation can be simultaneously achieved once the detection is finished. In the developed system, the breast pectoral muscle, which is usually shown in the mediolateral oblique (MLO) view, is firstly removed by an improved Deeplabv3+ segmentation module (Yu et al., 2022). We then proposed a multiple-level thresholding method to perform coarse segmentation of breast mass. The proposed multiple-level thresholding method first segments the breast tissues by the averaged pixel intensity of the breast tissues. After analysing the region properties of the connected components (ConCs) in the segmented images, the ConCs with large areas and the corresponding pixels in breast images are selected for further fine-grained segmentation based on the averaged pixel intensity of the selected pixels. The segmentation procedure stops only when no big enough ConCs are found in the segmented images. The segmentation results by different levels of thresholding are then combined to form the final segmentation results. To depress the noisy ConCs in the segmented images, an area opening operation, which eliminates the dot-like ConCs with a small area, is applied. In the final segmented images, each individual ConC and corresponding image patch is then extracted for breast mass and tissue classification, which is implemented by retraining the state-of-the-art deep learning models. The image patches that are classified as breast mass and corresponding ConC patches are taken as the coarse detection and segmentation results. Non-maximum Suppression (NMS) algorithm is applied to refine the results by suppressing the low-scored patches. The image patches that survived all stages can then be taken as the true breast masses, and then the patch-level detection results can be obtained. Furthermore, the segmentation result can be retrieved from the ConC patches, while the accurate detection result can be refined by the bounding boxes of the ConC patches. The main contributions of this paper can be concluded as follows:

1. We proposed a novel patch-based CAD system for efficient breast mass detection in mammography. Instead of deploying a deep learning object detection framework, we converted the detection problem into a single classification task and therefore saved overall computational cost. Compared to detection frameworks for common objects, the proposed framework is more friendly for training as only deep classifiers are introduced. Experimental results on two public datasets showed comparable performance to the benchmarks.
2. The proposed system is of high robustness that can be applied to mammography images with varied breast densities. In mammography images with dense breast tissues, it is a challenging task to detect breast mass. The situation is more complicated when the breast density of mammography varies from one image to another. However, our proposed multiple-level thresholding method can easily cope with varied breast density with a few predefined parameters. Therefore, the proposed algorithm is of high generality and flexibility.
3. We attempted a coarse breast mass segmentation at the same time as implementing breast mass detection. By introducing the multiple-level thresholding segmentation method, the coarse segmentation of breast mass can be obtained once the breast mass candidate patches are determined as a breast mass. While the segmentation can be refined, it could lead to new strategies for simultaneous breast mass detection and segmentation.

The remainder of this paper is arranged as follows. In Section 2, we will briefly revisit the related works in recent years. Then we will introduce the proposed pipeline in Section 3, followed by the experiments in Section 4, where we will introduce the details of the datasets used in this research, the setting of the experiments and the results. We will then discuss some issues regarding the problem in the experiment in Section 5. Finally, we end this paper with the conclusion and future work in Section 6.

## 2 Related works

Breast mass detection is an important module in mammogram analysis systems as it provides the region of interest (ROI) for further analysis. Given the importance of breast mass detection, there are numerous meaningful attempts toward it from the perspective of traditional and deep learning-based methods(Wang et al., 2018; Cao et al., 2021; Sun et al., 2021). In the work (Wang et al., 2018), Wang et al. proposed to integrate Gestalt psychology into breast mass detection tasks. The proposed framework is comprised of sensation integration, semantic integration, and verification. The proposed method reported a 93.84% detection sensitivity on the Digital Database for Screening Mammography (DDSM) at an FPI of 2.21(Heath et al., 1998). However, the false positive rate can be further reduced. In another deep learning-based method (Cao et al., 2021), an anchor-free architecture was developed. In the developed model, the contrast between breast mass and surrounding tissues is enhanced based on adaptive histogram equalization. The authors then transferred a one-stage detection network called FSAF, which is an anchor-free model, for the detection task here (Zhu et al., 2019). The authors reported the recall rate as 0.943 on DDSM at a 0.599 false positive rate. In another work (Aly et al., 2021), Aly et al. proposed to deploy You Only Look Once (YOLO) for breast mass detection and classification in INbreast mammograms. For comparison, the authors also deployed ResNet and Inception as feature extractors for classification performance against YOLO. The reported results showed that 89.4% of the masses in INbreast can be detected while an average precision of 89.4% and 94.2% were reported for benign and malignant masses classification. In another recent work(Sun et al., 2021), Sun et al. proposed to combine traditional template matching with deep learning. In the proposed method, ROIs are determined by scanning the mammographic images from top to bottom and then left to right via a morphology method that can transform brighter regions into circular-like areas. Deep convolutional neural networks (CNNs) are then trained to classify the ROIs into breast mass and breast background tissues. The reported detection results on DDSM were 86.82% sensitivity with 0.53 FPI. However, the robustness of the proposed method is poor as a minor change in the mammographic images, such as the intensity, would lead to detection failure.

Segmentation can also help breast cancer detection and diagnosis by introducing extra information(Min et al., 2020; Yan et al., 2021; Su et al., 2022). In the work (Min et al., 2020), simultaneous mass detection and segmentation are achieved by introducing a novel deep CNN model called Mask R-CNN that can simultaneously detect and segment objects of interest in images. A two-staged mass detection and segmentation framework can be found in (Yan et al., 2021) where breast masses are detected by a multi-scale fusion-based method and then are segmented via an improved version of UNet. Another transformer-based YOLO framework was introduced in the work (Su et al., 2022) that showed a true positive rate of 95.7% on breast mass detection on CBIS-DDSM. There are also some meaningful attempts at reducing computational costs for medical image analysis (Mukherjee et al., 2019; Zamzmi et al., 2021). In the work (Mukherjee et al., 2019), the authors proposed a segmentationfree method for automatic white matter injury detection in preterm infants. A linear maximally stable extremal regions algorithm with efficiency was first applied to detect the ventricles as blobs. Tissues that adjoin the blobs were identified via brainbackground boundary and a reference contour equidistant from the blobs. These tissues were assumed to follow a normal distribution of the grey-value intensity, and then outlier intensities were labelled as potential white matter injury, which was reconfirmed through the following heuristics. The proposed method is quite inspiring in that it can be transferred to similar scenarios such as breast mass detection, where the linear maximally stable extremal regions algorithm might be helpful in distinguishing breast mass from breast tissue.

In conclusion, the exploration of developing simultaneous breast mass detection is still quite limited, while some proposed methods heavily rely on computational resources for segmentation. Therefore, we proposed a novel framework for these two tasks. After an automatic search of proper thresholding values for segmentation, breast images are first segmented. We then extracted breast tissue patches regarding the ConCs in the segmented images for breast mass and background classification. The classification result is then refined by introducing Non-Maximum-Suppression (NMS). The advantages of the proposed framework include high performance and high robustness as we evaluated the proposed framework on two public datasets with promising results obtained.

## 3 Methodology

**Fig. 1:**
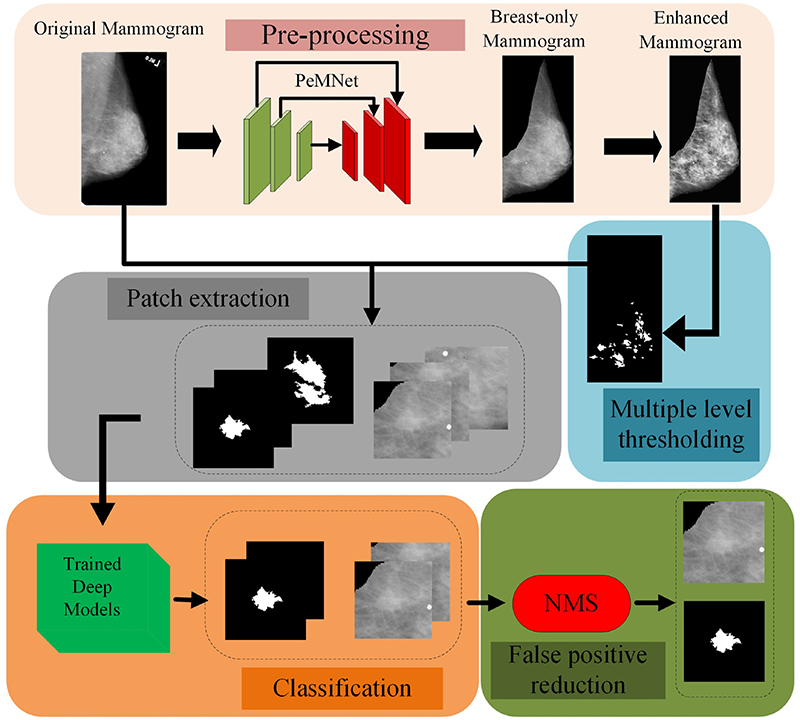
The overview of the proposed framework, which includes pre-processing, multiple-level thresholding, and breast mass detection, where breast mass detection is composed of path extraction, classification and false positive reduction.

In this section, we will introduce each module in the proposed framework, including pre-processing, multiple-level segmentation, and breast mass detection, where breast mass detection can be further divided into breast mass patch extraction, breast mass classification, and false positive reduction. In the pre-processing module, we mainly remove the breast pectoral muscle and enhance the contrast of the breast-only image. The multiple-level thresholding segmentation is then performed on the pectoral muscle removed and contrast-enhanced images. The varied thresholding values are applied to binarize the breast region into different ConCs, where corresponding breast tissue patches are extracted for mass detection. In the breast mass detection stage, deep learning models are transferred and retrained for breast mass and tissue classification. To further reduce the false positives after classification, we then applied the NMS algorithm and took breast mass patches that survived all stages as true breast mass. An overview of the proposed framework can be seen in Fig. 1.

### 3.1 Pre-processing

Pre-processing plays a key role in reducing computational costs and improving image quality. In mammograms, breasts only appear in a limited area, so breast region extraction alone could greatly benefit the following modules by shrinking the image size. In this paper, we propose to remove the breast pectoral muscle and enhance the resultant images for the following reasons. One is that the breast pectoral muscle will affect our intensity-based segmentation method as the intensity of the muscle is of high similarity to that of a true breast mass. Also, the size of the breast region can be further narrowed down once we have the pectoral muscle removed and therefore, we can reduce the overall computational cost. The reason why we enhance the contrast of images is that medical images usually suffer from low contrast and intensity inhomogeneity. As a result, image contrast enhancement would greatly mitigate the situation.

**Fig. 2:**
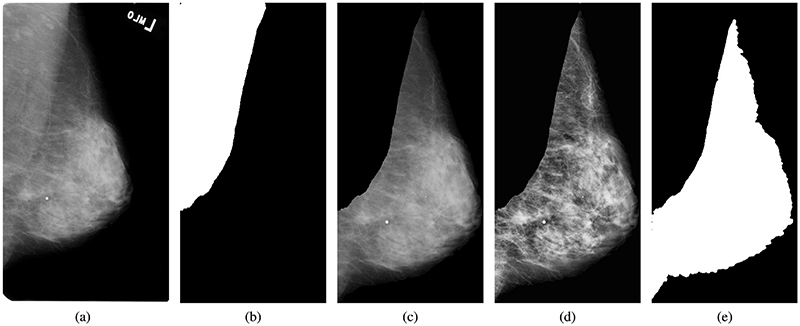
Pre-processing. **(a)** Original mammogram. **(b)** Segmented Pectoral muscle. **(c)** Breast-only image. **(d)** Contrast-enhanced breast-only image by CLAHE with clip limit as 0.02. **(e)** Obtained breast mask.

A mammogram must fall into one of four views, including a leftside mediolateral oblique (LMLO), a left-side craniocaudal (LCC), a right-side mediolateral oblique (RMLO) and a right-side craniocau-dal (RCC). However, the pectoral muscle usually appears in MLO view, while little or no pectoral muscle is shown in CC view mammograms. For pectoral muscle removal, we deployed a novel deep segmentation model called PeMNet in the work (Yu et al., 2022). After pectoral removal, the breast-only images in the MLO view are then enhanced by a classic method called contrast-limited adaptive histogram equalization. The mammograms in CC views, however, are directly applied with the contrast-enhancement method. One pre-processing example is given in Fig. 2. As can be seen, the pectoral muscle has been successfully segmented and removed. Compared to Fig. 2a, the number of interested pixels in the breast region has been greatly reduced. We then performed a classical contrast-enhancement method called Contrast Limit Adaptive Histogram Enhancement (CLAHE) on the breast-only images. After we segmented the pectoral muscle, the breast mask can be obtained from the segmentation results by simply setting the pixel corresponding to breast pixels to ones. As can be seen from Fig. 2, the pectoral muscle has been successfully removed while the contrast of the resultant image has been improved. Therefore, we believe our pre-processing procedures are effective and helpful in reducing overall computational costs while improving the quality of breast images.

### 3.2 Multiple-level thresholding segmentation

**Fig. 3:**
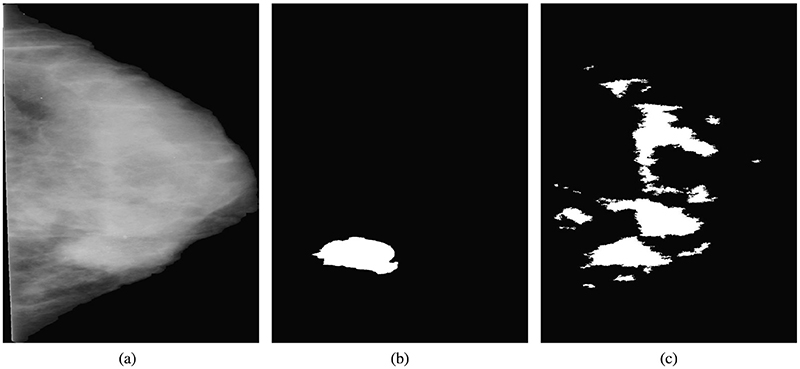
Multiple-level thresholding. **(a)** Original mammogram. **(b)** Pixel-level ground truth of breast mass. **(c)** Segmentation results by multiple-level thresholding. There are some segments in the segmented results due to the remaining pectoral muscle.

After pectoral muscle removal, the breast mass turns out to be the area of highest intensity if there is any breast mass in the presence of the mammogram. Based on this assumption, we deployed a multiple-level threshing segmentation algorithm, which can be divided into coarse and fine-grain segmentation. Given the preprocessed breast image *I* ∈ ℝ^*H*x*W*x3^, where *H* and W stand for the height and width of the image, respectively. Correspondingly, as mentioned before, we can have the breast mask **BMask** ∈ ℝ^*H*×*W*x3^ for the breast region, which indicates the breast-only region by 1 and the non-breast region by 0. We first calculated the mean intensity of the breast region and segmented the breast image based on the obtained value. As a result, the coarse segmentation result is obtained and then labelled into different ConCs. For each oversized ConC, a fine-grained segmentation is carried out. To determine whether a ConC is oversized or not, we predefined a fixed value **Area**, and the ConC is believed to be oversized if its area is greater than **Area**. Fine-grained segmentation proceeds when there are still oversized ConCs in the segmented images. Finally, all segmentation results are aggregated to form the final segmentation result. The detailed algorithm is shown in Algorithm 1. The detailed intermediate results of the segmentation process can be found in Fig. 3. Note that we analyzed the region properties of the ConCs in the segmentation result and then removed noisy ConCs such as segmentlike and the small dot-like ConCs, which can be seen in Fig. 3c.

Algorithm 1 **Multiple-level thresholding segmentation**

**Input** : Breast-only image ***I***, Breast mask ***BMask***

**Expected output**: Segmentation result Output *O^FS^*

   **Step 1: Calculate mean intensity** *M_R_*

(1) $$M_{R}=\frac{\sum \sum I\cdot BMask}{\sum \sum \;\text{BMask}\;}$$

   **Step 2: Coarse segmentation and labeling**

(2) $$O^{CS^{\prime }}(i,j)=\{\begin{array}{ll} 1, & \;\text{if}\;I(\text{i},\text{j})\geq \alpha M_{R}\\ 0, & \;\text{if}\;I(\text{i},\text{j})<\alpha M_{R} \end{array}$$

$O^{CS^{\prime }}\in {\left\{0,1\right\}}^{H\times W}$ stands for the coarse segmentation result that only comprises zero or one. $O^{CS^{\prime }}$ is then labeled into *n* ConCs regarding a scaled mean intensity, where *α* is the weight. So that $O^{CS^{\prime }}$can be denoted by the aggregation of ConCs as:

(3) $$O^{CS^{\prime }}=O_{1}^{CS^{\prime }}\cup O_{2}^{CS^{\prime }}\cup \cdots O_{n}^{CS^{\prime }}$$

where $O_{1}^{CS^{\prime }},\cdots ,O_{n}^{CS^{\prime }}\in {\left\{0,1\right\}}^{H\times W}$is a positive scale factor. To depress the noisy ConCs in the segmentation result, we performed an area opening operation.

**for***i* ⟵ 1 to *n***do**

   if $\sum \sum O_{i}^{CS'}>Area^{CS}$**then**$O^{CS}=O^{CS}\cup O_{i}{\;}^{CS^{'}}$ where $O_{1}^{CS^{\prime }},\cdots ,O_{n}^{CS^{\prime }}\in {\left\{0,1\right\}}^{H\times W},\alpha$, and *Area^cs^* is a small predefined threshold value. So that

(4) $$O^{CS}=O_{1}^{CS^{\prime }}\cup O_{2}^{CS^{\prime }}\cup \cdots O_{m}^{CS^{\prime }}(1\leq m\leq n)$$

For simplicity, we integrated the process of **Step 1** and **Step 2** by the function:

(5) $$O^{CS}=\text{CoSeg}(I,BMask)$$

   **Step 3: Fine-grain segmentation**

**for***i* ⟵ 1 to *m***do**

   if $\sum \sum O_{i}^{CS'}\geq$***Area then***

(6) $$O_{i}^{FS}=\text{CoSeg}(I,O_{i}^{CS})$$

**else**

(7) $$O_{i}^{FS}=O_{i}^{CS}$$

where ***Area*** is a predefined threshold value for the identification of ConCs with a large area. $O_{j}^{FS}\in {\left\{0,1\right\}}^{H\times W}$ stands for the fine-grained segmentation results corresponding to the ConC labelled as *j*. By aggregating all fine-grained segmentation results, we can then obtain the final segmentation result. The process can be shown as:

(8) $$O^{FS}=O_{1}^{FS}\cup O_{2}^{FS}\cup \cdots O_{m}^{FS}$$

### 3.3 Breast mass detection

Breast mass detection can be subdivided into three steps, including image patch extraction, classification and false positive reduction. Based on the segmentation results, corresponding patches from the processed breast-only images can be extracted regarding the bounding boxes. We extracted square image patches as deep learning models usually take square images as input. Detailed patch extraction procedures can be seen in Algorithm 2.

Algorithm 2 **Image patch extraction.**

**Step 1: Initialization**

1) Label the ConCs in the segmentation results into 1, …, *m*;
2) Obtain the bounding box *BBox*(*cx*, *cy*, *width*, *height*)$\in {\mathbb{R}}^{4\times m}$ of $O_{i}^{FS}(i=1\cdots m)$, where *cx*, *cy*, *width*, *height* stands for the horizontal centroid, vertical centroid, width and height of the ConCs, respectively.
3) Initialize default image patch size as *Size*;
4) Initialize the expected output location *EBox*(*cx*, *cy*, *width*, *width*)$\in {\mathbb{R}}^{4\times m}$.

   **Step 2: Location adjustment**

**for** *i* ← 1 to *m* **do**

   *Size_i_* = *max*(*BBox*_*i*_(*width*), *BBox*_*i*_(*height*), *Size*)

   *EBox*_*i*_(*cx*) = *min*(*max*(l +

   0.5*Size*_*i*_, *BBox*_*i*_(*cx*)), *W* – 0.5*Size*_*i*_)

   *EBoX*_*i*_(*cy*) =

   *min*(*max*(l+0.5*Size*_*i*_, *BBox*_*i*_(*cy*)), *H* – 0.5*Size*_*i*_)

   *EBox*_*i*_ (*width*), *EBox*_*i*_(*height*) = *Size*_*i*_

where *max*(·) and *min*(·) stand for maximum and minimum operations correspondingly.

   **Step 3: Patch extraction**

*BP*_*i*_ = **I**(*EBox*_*i*_(*cx*) – 0.5 *EBox*_*i*_(*width*): *EBox*_*i*_(*cx*) + 0.5 *EBox*_*i*_(*width*) – *EBox*_*i*_(*cy*) + 0.5 *EBox*_*i*_(*height*))

where *BP*_*i*_ indicates the image patch corresponding to the ConC labelled as *i*, where each individual ConC patch $OP_{i}^{FS}$ can be extracted by: $OP_{i}^{FS}$ = $OP_{i}^{FS}$ (*EBox*_*i*_(*cx*) – 0.5 *EBox*_*i*_(*width*): *EBox*_*i*_(*cx*) + 0.5 *EBox*_*i*_(*width*) – 0.5*EBox_i_* (*heights*):*EBox*_*i*_(*cy*) + 0.5 *EBox*_*i*_(*height*))

**Fig. 4:**
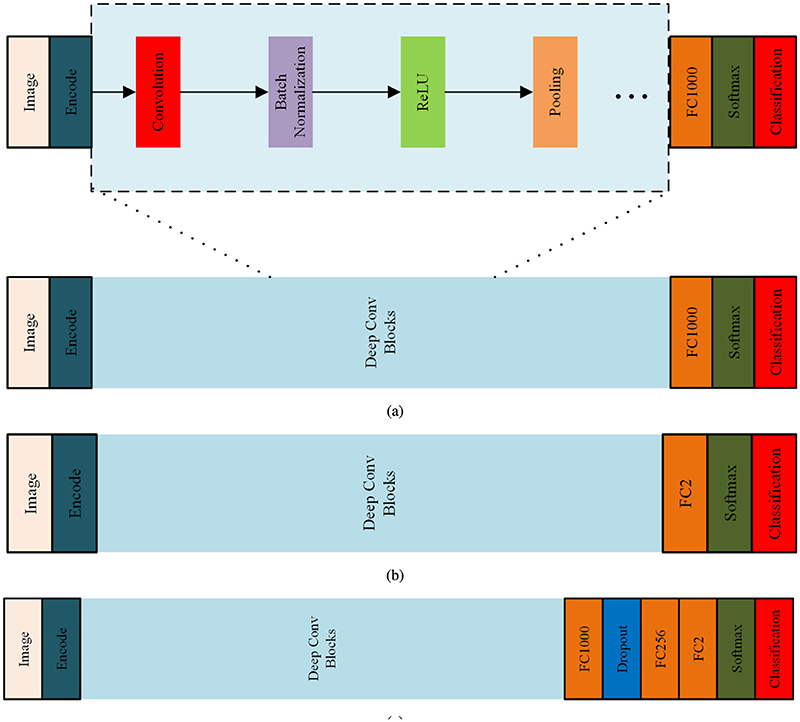
Architectures of deep learning models. **(a)** Original deep learning models. **(b)** Adapted deep learning models by simply replacing the last fully connected layers.**(c)** Adapted deep learning models by adding extra layers. FCX stands for a fully connected layer with X-dimensional output. The dropout layer is introduced to prevent the overfitting issue while FC256 is introduced to prevent significant information lost from FC1000 to FC2.

**Fig. 5:**
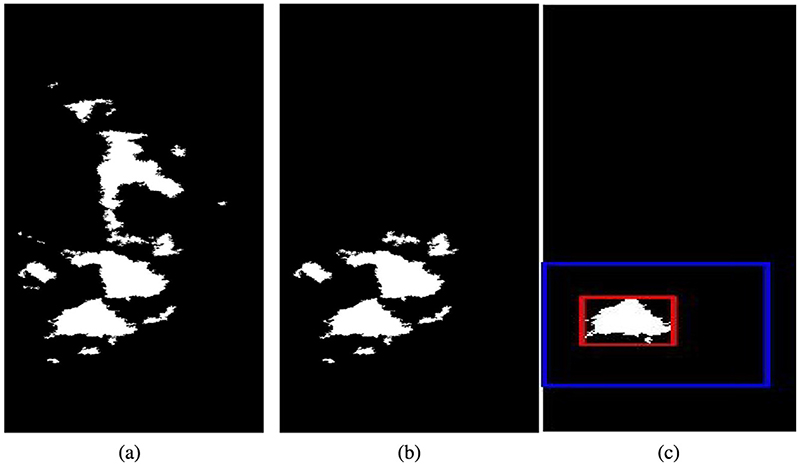
Patch classification and Non-maximum suppression. **(a)** Post segmented results. **(b)** ConCs correspond to patches that are classified as breast mass by deep learning models. **(c)** The detection result. The blue bounding box indicates the patch location in the image while the red one indicates the refined bounding box for accurate detection result.

The architectures of deep learning models before adaptation and after can be seen in Fig. 4. We deployed the state-of-the-art deep learning models that were pre-trained on the ImageNet dataset for breast mass and tissue (Simonyan and Zisserman,2014; He et al., 2016; Huang et al., 2017; Szegedy et al., 2017). Those deep learning models have achieved dominating performance on 1,000 categories of classification compared to other methods, which can be seen in Fig. 4a. In deep learning models, a encode layer is responsible for adjusting the size of input images to the input size requirement. The main components in deep learning models are deep blocks that comprise stacks of convolution layers, normalization layers, and activation layers, i.e. ReLU layer, pooling layers in top of Fig. 4a. The features generated from deep conv blocks are fed to a fully connected layer, which is responsible for mapping the learnt features into target space for the classification tasks. For efficient deployment, we transferred these models to our classification task by introducing minimal changes. There are two most straightforward ways to adapt those deep learning models for our classification task here. The first one is to simply replace the original fully connected layer with the expected fully connected layer (Ali et al., 2021), which is shown in Fig. 4b. We aimed at a two-class classification task here and we, therefore, replace the original fully connected layer with a two-neuron fully connected layer. And the second one is to add more layers after the final fully connected layers for desired classification task as is shown in Fig. 4c. To prevent significant information loss, similar to the works in (Xiang et al., 2020; Yu et al.,2021), we added a new fully connected layer with 256-dimensional output. Also, we added a dropout layer at the dropout rate of 0.5. After breast mass classification, numerous overlapping image patches will be predicted as masses. To solve this and reduce false positives, we introduced the NMS algorithm, which can be seen in Algorithm 3. An example can be seen in Fig. 5. As can be seen, only patches containing breast mass are kept in Fig. 5b where there are multiple detection results. By performing the proposed NMS, the detection results combine into a single detection result while the FPI reduces simultaneously.

Algorithm 3 **Non-maximum Suppression.**

**Input** : Predicted scores: *Scores* in ${\mathbb{R}}^{s}$, ConC patches: $OP_{i}^{FS}(1\leq i\leq s)$ Locations: *location(cx, cy, width, height*) $location(cx,cy,width,height)\in {\mathbb{R}}^{4\times s}$

**Expected output**: Refined scores: $scores_{R}\in {\mathbb{R}}^{s'}(s'\leq s)$, Refined locations:$location_{R}(cx,cy,width,height)\in {\mathbb{R}}^{4\times s'}$

**for***i* ⟵ 1 to *s* – 1 **do**

   **for***j* ⟵ *i* + 1 to *s***do**

      **if***IoU*(i,j) > ***Rate* then**

         **if***Scores*(i)! = 0 **then**

          **if**$Scores(j).\sum \sum OP_{j}^{FS}\leq Scores(i).\sum \sum OP_{i}^{FS}$**then**

          *Scores*(*j*) = 0;

          **else**

          *Scores*(*i*) = 0;

where *IoU*(*i,j*) denotes the area of intersection of union between *i_th_* and *j_th_* image patch and the ***Rate*** is the intersection rate.

*count* = 1;

**for***i* ⟵ 1 *to s***do**

   **if***Scores*(*i*)*!*= 0 **then**

      *Scores_R_*(*count*) = *Scores*(*i*)

      *localion_R_*(*cx_count_*, *cy_count_*, *width_count_*, *height_count_*)

      *location*(cx_i_, cy_i_, *width*_i_ *height*_i_)

      *count* = *count* + 1

### 3.4 Model training and inference

In the training stage, patch classification is the only module that requires training as no learnable parameters are found in other modules. When training the deep CNN models, breast mass patches in the training set are directly extracted regarding the bounding boxes and are fed to the CNN models. The trained deep CNNs tend to recognize the breast mass patches that appeared in the training set before. The overall evaluation of the detection framework on the train set doesn't make too much sense. Instead, the overall evaluation of the testing set relies on some predefined parameters, where the performance of the detection framework may vary slightly due to these parameters. We, therefore, will explore the possible combinations of these parameters in the model inference stage instead of fixing them in the training stage. When inferring, the full mammogram in the testing set is preprocessed regarding the pre-processing module that extracts the breast region and removes the pectoral muscle. Then multiplelevel thresholding is applied to generate segmentation results for breast mass. According to the location information of the ConCs in the segmentation result, breast patches are extracted and then classified by the trained deep CNNs. The patches that are classified as breast mass are aggregated for false positive reduction by NMS. However, patches in some mammograms may fail to be classified as breast mass due to the difference in patch extraction from the training set and the testing set and the complexity of the mammogram. Note that breast mass patches in the training set are extracted regarding the true location information, while the patches in the testing set are extracted based on the segmentation results. Considering this, we take *t* patches with the top-ranked scores as the breast mass candidates for false positive reduction when no patches are classified as breast mass in the mammogram. We then consider it a successful detection of breast mass when the overlapping rate between the true bounding box and predicted bounding box is no less than 0.2.

## 4 Experiment

In this section, we will briefly introduce the datasets involved in this research. Later on, we will introduce the setting of parameters in the experiment. The key part of the proposed framework is the performance of the breast mass classification model. So, we will present the performance of the adapted deep learning models before we move to the detection results on two public datasets. We then finish this section with the method comparison, where we will compare our method with the state-of-the-art methods.

### 4.1 Datasets

**Fig. 6:**
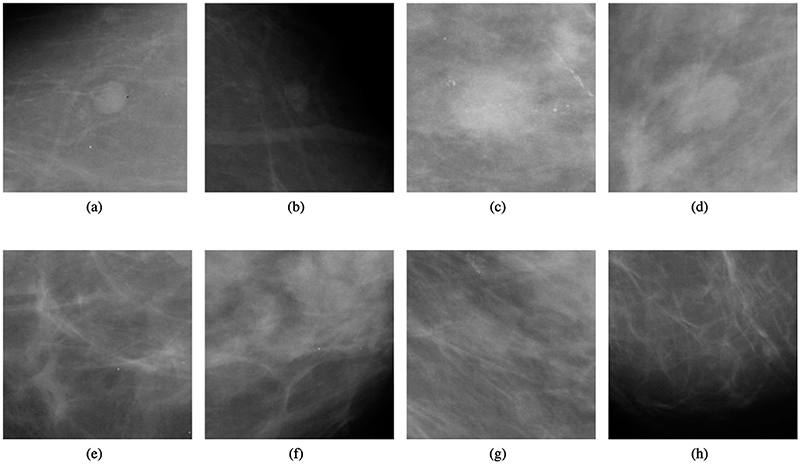
Manually extracted patches from the training set. **(a)** Benign mass with smooth shape. **(b)** Benign mass close to breast boundary. **(c)** Malignant mass with unclear border. **(d)** Irregular malignant mass. **(e)** to **(h)** refer to normal breast tissue examples.

In this research, we conducted our experiments on two public datasets, i.e., CBIS-DDSM and INbreast (Lee et al., 2017; Moreira et al., 2012), both of which provide pixel-level annotated ground truth. More importantly, all of the mammograms from the two datasets may have different breast densities that may cause breast mass detection failure. The height and width of the mammograms from the two datasets are usually more than 4000 pixels and 2000 pixels, respectively. We used the training set of CBIS-DDSM for model training and the testing set for evaluation while we directly evaluated the performance of the proposed framework on INbreast dataset without any further adaption. When training the models, we manually extracted mass patches and breast tissue patches from the training set of CBIS-DDSM, where the breast tissue patches have no overlaps with the breast mass. We obtain the breast tissue patches through the sliding window technique while breast mass patches are extracted directly regarding the given annotations. The breast tissue patches are extracted only when there is no intersection between the breast mass patches found. By doing so, the number of breast tissue patches greatly outnumbers that of the breast mass patches. We then applied data augmentation to the breast mass patches while randomly selecting the same number of breast tissue patches. The applied data augmentation methods include flipping upside down, flipping left to right, flipping upside down and then flipping left to right, contrast enhancement by CLAHE with the clip limit of 0.02, random scaling from 1 to 1.2, rotation clockwise by 90 degrees, and rotation counter-clockwise by 90 degrees. By aggregating all augmented images and the original image in the training set, the augmented training set was scaled to eight times of the original size. Similarly, we extracted the breast mass and tissue patches from the test set for evaluation of the deep learning models in the same way. Same here in the testing set, breast tissue patches greatly outnumbered breast mass patches, which will harm the evaluation metrics. Therefore, we created an augmented testing set only for the evaluation of deep learning models. The detailed composition of the dataset for deep learning model training can be seen in Table 1. Note that for overall detection performance evaluation, we applied to proposed patch extraction method to the testing set instead. Some extracted breast mass patches and tissues are shown in Fig. 6. As can be seen, the breast mass patches may vary in size, shape, and location.

In INbreast dataset, there are in total of 410 images while only 107 images are confirmed with mass, which is called the validation set in this study. Therefore, we directly performed the proposed framework on those mammograms for model evaluation.

### 4.2 Experiment settings

The overall performance of the proposed framework is determined by some predefined key parameters and the classifier for breast mass classification. The predefined parameters, which are non-learnable, indirectly determine the performance of detection. The detailed descriptions of these parameters can be seen in Table 2.

When the value of α is greater than 1, the mean intensity is then scaled up and therefore, only pixels with higher intensity are kept in the segmentation results, which will impair the detection capability. Therefore, we set the value of α to be 0.8 by default to avoid segmenting breast mass into the background in the first stage of segmentation. The value of *Area^CS^* and *Area* determines the number of ConCs during segmentation. A larger *Area^CS^* tend to eliminate more noisy ConCs in the segmentation results. However, the true masses with a small area are also likely to be removed. Considering this, we set *Area^CS^* to be 200. On the contrary, the choice of *Area* is easier in that a large value is sufficient. By default, we set it to 50,000. *Width* controls the size of patches to be considered when extracting patches. A small *Width* is likely to provide more localized detection results with more false positives, while a larger *Width* may contribute to higher detection sensitivity as each image patch has a larger scope. The correlation between the value of *Width* and the detection performance remains to be explored, which will be shown later in the experiment. *Rate* is another parameter that will affect both detection sensitivity and the false positive rate of the framework. The relation between *Rate* and detection sensitivity will be revealed in the experiment. For t, we empirically set it to be 10 as it is not the key parameter that will significantly affect the detection sensitivity. In conclusion, we will determine the parameters, including *Width* and *Rate* via experiments in a later section.

Instead, the performance of the classifiers directly determined the performance of detection. In this work, the deep learning models used are VGG19, ResNet50, InceptionV3, DenseNet201, InceptionResnetv2, and EfficientNet (Tan and Le, 2019), as they are the most representative state-of-the-art deep CNN models. Some details of these models have been listed in Table 3, where the term FLOPs stands for floating point operations. As was mentioned before, we introduced two fully connected layers and one dropout layer to the top of the pre-trained deep learning models. So that the number of the introduced parameters is 1000×256 + 256×2 = 256, 512. Therefore, the numbers of the adapted deep learning models are the sum of the original numbers of parameters and 256, 512. The SPECTRE High-Performance Computing Facility at the University of Leicester with a 16 GB memory GPU is deployed for model training. The training parameters can be seen in Table 4, where SGDM stands for Stochastic Gradient Descent with Momentum.

### 4.3 Performance of patch extraction

We aimed at extracting all of the image patches containing breast mass via the proposed patch extraction method. So before we move to breast mass detection directly, we evaluated the performance of the proposed patch extraction method on breast mass extraction with varied parameters such as α and *Width*. A larger α means a higher threshold value so that fewer ConCs and image patches will be generated. However, the higher threshold value may also falsely segment the breast mass with low intensity into the background. As a result, the value of α should be carefully chosen. The *Width* determines the size of the extracted image patches so large values of *Width* seem to be more advantageous than small values of *Width*. Note that deep learning models usually require a fixed size of the input so image patches have to be resized to meet the input requirement. However, oversized image patches may suffer from significant information loss when they are resized to a much smaller size. This brought challenging classification situations to the deep learning models. Considering this, the values of *Width* should be carefully fine-tuned as well. We then varied the values of α and *Width* and recorded successful extraction on CBIS-DDSM in Table 5. By saying a successful extraction, we mean that the extracted patch contains at least half of the breast mass. As can be seen, the successful extraction rate increases along with *Width* when α is fixed. Also, the successful extraction rate decreases along with the increment of *Width* when *Width* is small. The situation is mitigated when *Width* increases while the highest successful extraction rate is achieved when *Width* = 299 and α = 1.1. Therefore, we consider larger *Width* is more advantageous, while the values of α should be fine-tuned after the determination of *Width*. Note that most of the paired a and *Width* can generate 100% successful extraction, so we skip the patch extraction validation on INbreast but move to the direct detection instead.

### 4.4 Model ablation for breast mass classification

To explore the best configuration of deep learning models towards breast mass classification, we then compared the performance of deep learning models trained under different configurations. For performance evaluation, we used metrics, including *Sensitivity*, *Specificity*, *Precision*, *F*1_score_ and *Accuracy*, Area under the Curve (AUC) of receiver operating characteristic curve. Given the predicted results, the conclusion can be described as True Positive (TP), True Negative(TN), False Positive (FP) and False Negative (FN). *Sensitivity*, which indicates the capability of the classifiers to spot true breast mass, can be denoted as:

(9) $$\text{Sensitivity}\;=\frac{TP}{TP+FN}$$

*Specificity*, *Precision*, *F*1_score_ and *Accuracy* can be expressed as:

(10) $$\;\text{Specificity}\;=\frac{TN}{TN+FP}$$

(11) $$\;\text{Precision}\;=\frac{TP}{TP+FP}$$

(12) $$F1_{\text{score}\;}=2\times \frac{\;\text{Precision}\;\times \;\text{Sensitivity}\;}{\;\text{Precision}\;+\;\text{Sensitivity}\;}$$

(13) $$\;\text{Accuracy}\;=\frac{TP+TN}{TP+FP+TN+FN}$$

**Fig. 7:**
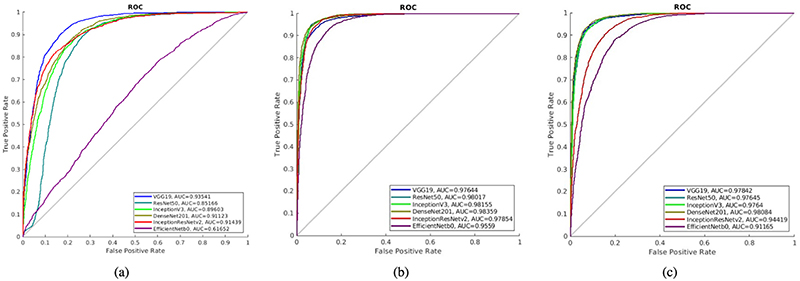
Performance of deep learning models on the adjusted testing set. **(a)** ROCs of deep learning models trained with the original training set. **(b)** ROCs of deep learning models with new layers introduced. **(c)** ROCs of deep learning models with only the final fully connected layer replaced. Deep learning models in **(b)** and **(c)** are trained with the adjusted training set.

To validate the effectiveness of data augmentation methods, we first trained different deep learning models on the original training set and tested the trained models on the adjusted testing set. The deep learning models here are the ones with several new layers introduced as was shown in Fig. 4c. The results are shown in Table 6 and the ROC curves are drawn in Fig. 7a. As can be seen from Table 6 and Fig. 7a, Vgg19 turns out to be the best model that achieved the overall accuracy of 0.87 on the adjusted testing set while obtaining an AUC of 0.94. We then train these models with the adjusted training set and have them validated on the adjusted testing set. The results are shown in Table 7 while the ROC curves are drawn in Fig. 7b.

**Fig. 8:**
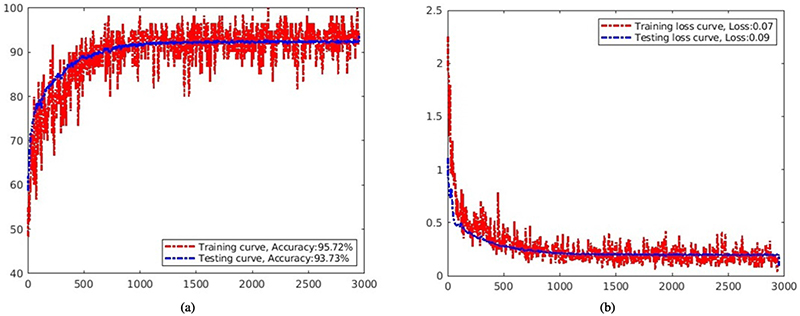
The learning curve of ResNet50. **(a)** Learning curves of ResNet50 on the adjusted dataset; Note that the model converges within 9 epochs due to the large volume of parameters and achieved 93.73% accuracy on the adjusted testing set. **(b) Loss curves of ResNet50 on the adjusted dataset, where the final training loss and testing loss reached 0.07 and 0.09, respectively**.

Compared to the deep models trained on the original training set, the performance of the deep models trained on the augmented training set has been improved greatly, which validated the effectiveness of data augmentation. As can be seen from Table 7, ResNet50 achieved the best performance amongst all networks in terms of Sensitivity and overall accuracy. However, the ROC curves indicate that DenseNet201 possesses the most powerful classification capability. Nevertheless, all networks enjoyed significant performance gains thanks to the diversity and increased number of images in the augmented training set. Fig. 8a and Fig. 8b in Fig. 8 indicate the learning curves and loss curves of ResNet50 on both the adjusted training set and the adjusted testing set, respectively.

To validate the necessity of introducing new layers, we then simply replaced the final fully connected layers with only two nodes as was shown in Fig. 4b. We trained these adapted models with the adjusted training set and then validated them on the adjusted testing set. The classification results are shown in Table 8 while the ROC curves are drawn in Fig. 7c. VGG19 turns out to be the best one in terms of overall accuracy and sensitivity. Compared to the models with new layers introduced, the models with the final fully connected layer replaced showed a slight decline in performance, which indicates the benefit of introducing new layers. Finally, we chose to deploy ResNet50, which was adapted by introducing new layers and was trained on the adjusted training set, as the classifier for the breast mass classification task given its model size and performance.

### 4.5 Detection results on CBIS-DDSM

In this section, we will explore the effect of the predefined parameters, including α,*Area*^CS^, *Width*, and *Rate* on the overall performance of the detection framework. We firstly checked the overall performance of the detection framework with varied α when *Width* = 299 as the best patch extraction performance is achieved when α = 1.1, and *Width* = 299. We then calculated the *p value* between the best detection result against other detection results in terms of sensitivity and FPI by carrying out t-tests. The null hypothesis (H0) is that the true difference between the sensitivity (FPI) of the groups is zero, which means we carried out the tests on sensitivity and FPI individually. We believe there is a significant difference if *p*-value is less than 0.05 and thus reject the null hypothesis. Without other specifications, *Area^CS^*, *Area*, and *t* are set to be 300, 50,000, and 8, respectively. The results can be seen in Table 9, where Sensitivity and FPI stand for the number of detected breast masses out of all masses and the false positive per image, respectively. *p – value_Sen_* and *p – value_FPI_* are the *p*-*values* of sensitivity and FPI, respectively. As can be seen, the best detection performance is achieved when α is 0.6. Also, the detection performance deteriorates along the increase of α. However, the *p-values* of sensitivity and FPI indicate that there is no significant between them. As a result, α doesn’t seem to be the key factor that influences the detection performance when *Width*, *Rate* are fixed. Based on the detection results, we believe a lower value of α is more beneficial to the overall detection performance. And in the experiment later on, we empirically set α to be 0.6. We then varied *Width* from 129 to 299 while keeping the *Rate* to 0.3. The detection results can be seen in Table 10, where ≪means significantly less than.

**Fig. 9:**
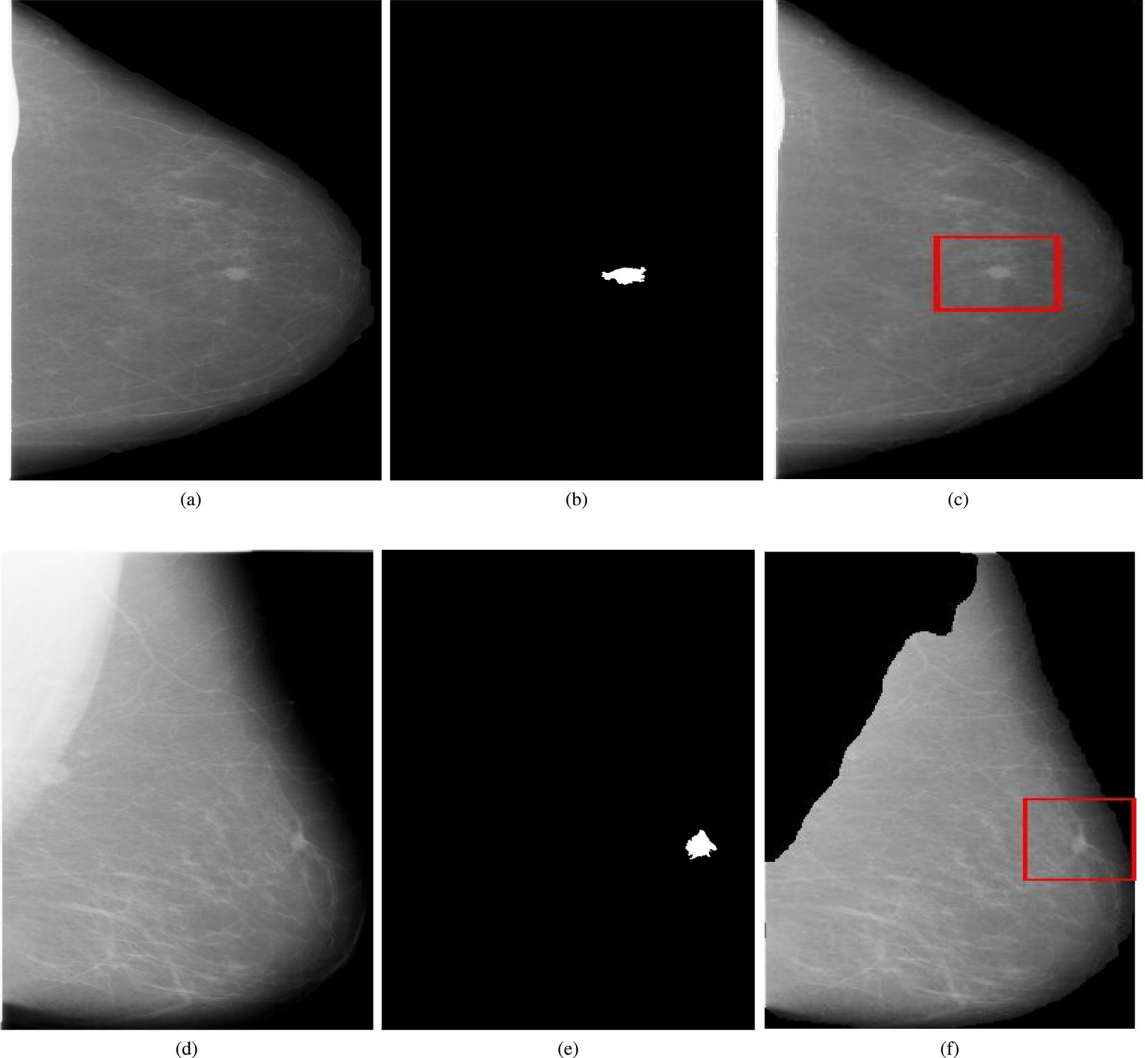
Detection examples from CBIS-DDSM when *Width* = 224 and *α* = 0.8. **(a)** Pre-Processed CC view breast image. **(b)** Pixel-level ground truth of breast mass for **(a)**. **(c)** Patch-level detection result for **(a)**: *Sensitivity* = 1, *FPI* = 0. **(d)** Original MLO view breast image. **(e)** Pixel-level ground truth of breast mass for **(d)**. **(f)** Patch-level detection results on pre-processed **(d)**: *Sensitivity* = 1, *FPI* = 0.

As can be seen from Table 10, the overall detection performance increases along with *Width*. The sensitivity increased from 70.06% to 83.33% when *Width* increased from 199 to 299. The FPI, on the contrary, decreases from 4.77 to 1.44. Combining the *p* – *values* of sensitivity and FPI together, the frameworks with varied *Width* showed significant differences in detection performance. As a consequence, we believe *Width* seems to be one of the key factors for detection performance. And a relatively large value of *Width* is more desirable, so we fixed *Width* to 299 for later experiments on the CBIS-DDSM dataset. Therefore, we then varied *Rate*, where the results can be seen in Table 11. As can be seen, a larger *Rate* leads to a higher detection sensitivity while producing a larger FPI at the same time. Nevertheless, the best detection performance is achieved with a sensitivity of 87.29% at 2.86 FPI. The statistical analysis indicates that detection performance is saturated when *Rate* reaches 0.7 and beyond. Nevertheless, *Rate* can be considered as another key factor that affects the overall detection performance. As a result, we may conclude that the overall detection performance can be improved via a lower value of α and a larger value of *Rate* when a larger *Width* is chosen. Some detection examples can be seen in Fig. 9. As can be seen from Fig. 9c and Fig. 9f, the breast masses have been accurately located, and therefore, further analysis such as mass classification can be deployed based on the patch-level detection results.

### 4.6 Detection results on INbreast

To further validate the proposed framework, we also tested the framework on INbreast dataset. Note that no more fine-tuning procedures are carried out for adaptation. The deep learning models are directly applied for breast mass and tissue classification and the same to the other following modules. Again, we set *Area*^CS^, *Area*, and *t* to 300, 50,000, and 8. Similarly, we then varied the values of α, *Width* and *Rate*. The detection results obtained via grid search can be seen in Table 12 when we fix α to be 0.6 while varying *Width* and *Rate*. Based on the sensitivity and FPI, the paired *P_S_* and *P_F_* on the last column justify the impact of *Rate* to the overall detection performance while counterpart on the bottom of the table indicates the influence of varied *Width*. The *p*-*values* are obtained via a two-way analysis of variance (ANOVA) test. The same conclusion can be drawn here that a larger *Width* leads to a higher sensitivity while a higher *Rate* produces a higher FPI. As a result, the best detection performance is achieved with a sensitivity of 95.41% and FPI of 1.69 when *Width* = 224 and *Rate*=0.5. The statistical analysis indicates that both *Rate* and *Width* collaboratively influence the detection performance when a is 0.6. We then repeated the experiment but adjusted α to 0.7 and the detection results can be seen in Table 13, where the best detection performance is achieved with the sensitivity of 96.33% and the FPI at 1.29 when *Width* = 256 and *Rate*=0.6. The impact of *Width* may not be significant given that the *p* – *value* of sensitivity is 0.28. Nevertheless, the *p*-*values* show that the overall detection performance is quite different by combining the *p-values* of sensitivity and FPI together.

**Fig. 10:**
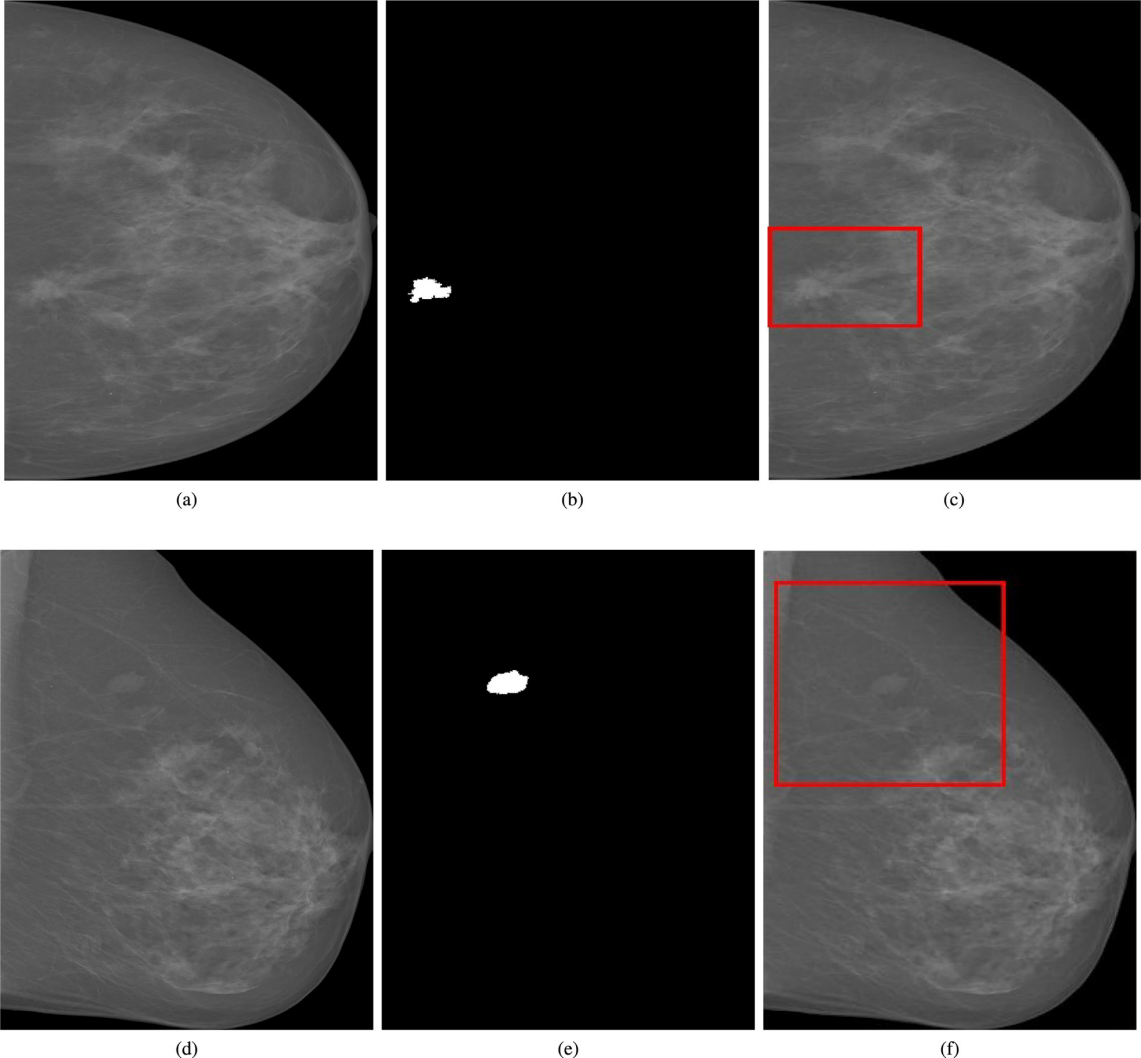
Detection examples from INbreast dataset when *Width* = 199 and *α* = 0.8. **(a)** Pre-Processed CC view breast image. **(b)** Pixel-level ground truth of breast mass for **(a)**. **(c)** Patch-level detection result for **(a)**: *Sensitivity* = 1, *FPI* = 0. **(d)** Another Pre-Processed CC view breast image. **(e)** Pixel-level ground truth of breast mass for **(d)**. **(f)** Patchlevel detection results on pre-processed **(e)**: *Sensitivity* = 1, *FPI* = 0.

We then set the value of α to 0.8 for optimal parameter exploration, which can be seen in Table 14. The best detection performance is achieved with a sensitivity of 94.50% and FPI of 1.53 when *Width* = 224 and *Rate*=0.5. The statistical analysis showed that sensitivities and FPIs from different groups are significantly different. Therefore, the conclusion that *Rate* and *Width* are two key factors is supported while α should be a low value. Compared to the detection sensitivity on the test set of CBIS-DDSM, the detection sensitivity on INbreast seem to be much higher, with the best one of 96.33 at the FPI only of 1.29. Also, it is interesting that the averaged FPI on INbreast dataset is much lower than that of CBIS-DDSM. The main reason could be the quality of the images, as images from INbreast dataset are of higher quality than the images from CBIS-DDSM. Some detection examples can be found in Fig. 10.

### 4.7 Method comparison

We then compared our proposed method with the existing state-of-the-art methods, as can be seen in Table 15. Compared to methods that have been validated on CBIS-DDSM, our method is more advantageous as it has higher sensitivity in detecting true masses but with acceptable FPI. On INbreast dataset, our proposed method also showed comparable performance at the lowest cost on FPI. While some methods reported higher sensitivity values, these methods, however, were validated on the subset of INbreast with fewer images. Nevertheless, some of these methods still produced high FPIs. Given the size of the testing set and the performance of the proposed method, we believe our method is still quite competitive and sever as a handy tool for breast mass detection.

## 5 Discussion

There are a few issues we’d like to discuss here. The first is the necessity of repetitive thresholding in the proposed multiple-level thresholding algorithm. In the proposed algorithm, we repeated the thresholding procedure twice as breast masses and issues with similar intensities are likely to merge into large connected components. Therefore, a second thresholding procedure would help to distinguish them. The second is that the choice of some parameters that are not so crucial in the proposed algorithm is determined via trial and error. For example, in the choice of *Area^CS^* for noise depression, we found that the value of 300 turned out to be the best one that gives the best detection performance. Another issue is the necessity of selecting top-ranked *t* image patches for detection. When completing the experiments, we found that the detection performance was quite low if we simply pick the patches with predictive scores beyond a predefined value. Because most of the extracted breast masses were only partially located within the images, which makes those images challenging to be recognized. Considering this, we decided to include the top-ranked image patches to include breast mass candidates as many as possible. The false positive rate, however, is likely to increase due to the increase of candidates, which is then mitigated by introducing the non-maximum suppression algorithm.

## 6 Conclusion and future work

In this study, we developed a novel patch-based breast mass detection framework. In the developed framework, we deployed a novel multiple-level thresholding algorithm based on the nature of breast mass. After the multiple stages of segmentation, breast tissue patches that may contain breast masses are then extracted regarding the ConCs in the segmented images. The detection problem is then converted to a classification problem as the image patches can be simply classified as breast mass and breast tissue. By introducing the multiple-level thresholding algorithm, variations such as image intensity and size of breast mass can be self-adapted. Therefore, the overall robustness of the proposed framework is improved. The experiments on two public datasets further supported the effectiveness of the proposed framework. Moreover, the proposed framework can process mammography images fast thanks to the low-cost thresholding algorithm. As a result, we believe the proposed framework can be helpful for the clinical practice of breast mass detection for mammography images. However, there are still some limitations to this work. One is the performance of deep learning models for breast mass and tissue classification. Note that the best-performed model ResNet50 can still be improved for higher classification accuracy. So that detection sensitivity can be boosted while FPI can be reduced. Another is that the optimization methods can be improved from multiple perspectives such as the Another is that more accurate detection results can be obtained through corresponding ConCs. In the future, more work can be done to obtain more accurate detection results regarding the ConCs. Also, the segmentation algorithm can be improved to produce better segmentation results instead of the current coarse results. In conclusion, this work provided a novel yet straightforward strategy for breast mass detection and can serve as the basic work for future works.

## Figures and Tables

**Table 1:** CBIS-DDSM dataset composition.

Dataset	Masses patches	Negative patches	Total
Original training set	1,230	24,171	25,401
Adjusted training set	9,840	9,840	19,680
Original testing set	353	14,103	14,457
Adjusted testing set	2,824	2,824	5,648

**Table 2:** Predefined parameters.

Parameter	Located in	Definition
*α*	Algorithm 1	The factor that scales the threshold in coarse segmentation.
*Area^CS^*	Algorithm 1	The noise depression threshold.
*Area*	Algorithm 1	The threshold for the determination of ConCs with a large area.
*Width*	Algorithm 2	The size of the extracted image patches.
*Rate*	Algorithm 3	The intersection rate that controls the sensitivity of Non-maximum suppression.
*t*	Patch extraction in the inference phase	The number of selected patches when breast mass classification fails.

**Table 3:** Total number of parameters for deep learning models.

Names	Number of parameters of original models(Millions)	Input size to newly added FC layer	FC layer neurons	Total number of parameters(Millions)	Number of FLOPs(Gbits)
VGG19	**143.68**	1000	256 & 2	$+\frac{256*1000+256*2}{10^{6}}=143.94$	**19.80**
ResNet50	25.56	1000	256 & 2	25.82	4.25
InceptionV3	24.11	1000	256 & 2	24.37	6.13
DenseNet201	20.02	1000	256 & 2	20.28	4.37
InceptionResNetv2	56.11	1000	256 & 2	56.37	6.64
EfficientNetb0	5.30	1000	256 & 2	5.56	0.02

**Table 4:** Setting of hyper-parameters.

Parameters	Values
Maximum training epoch	9
Initial learning rate	10^−4^
Mini-batch size	60
Learning rate drop period	3
Learning rate drop rate	0.1
Optimization method	SGDM
Shuffle of the train set	Each epoch
Momentum	0.9

**Table 5:** Performance of patch extraction on CBIS-DDSM.

	Successful extraction rate(%)					
*α*	*Width* 129	156	199	224	256	299
0.8	88.70	92.94	97.46	98.31	98.59	98.59
0.9	88.14	93.22	96.61	98.31	98.59	99.51
1.0	84.75	90.96	97.18	98.02	98.87	99.44
1.1	85.31	91.24	97.18	98.59	99.15	**100**
1.2	80.79	87.85	95.48	98.02	98.87	99.44

**Table 6:** Performance of the deep learning models trained with original training set while being evaluated on the adjusted testing set. The bold indicates the best.

Models	Sensitivity	Specificity	Precision	F1_*score*_	Accuracy	AUC(95% confidence interval)
Vgg19	**0.83**	**0.91**	**0.90**	**0.87**	**0.87**	**0.9376~0.9466**
ResNet50	0.74	0.89	0.87	0.80	0.81	0.8445~0.8588
InceptionV3	0.76	0.89	0.87	0.81	0.82	0.8900~0.9020
DenseNet201	0.77	0.89	0.88	0.82	0.83	0.9057~0.9168
InceptionResNetv2	0.76	0.89	0.88	0.81	0.83	0.9090~0.9198
EfficientNetb0	0.53	0.64	0.59	0.56	0.58	0.6062~0.6268

**Table 7:** Classification performance of different deep learning models trained with the adjusted training set while being evaluated on the adjusted testing set.

Models	Sensitivity	Specificity	Precision	*F*1_*score*_	Accuracy	AUC(95% confidence interval)
Vgg19	0.90	0.94	0.94	0.92	0.92	0.9735~0.9793
ResNet50	**0.91**	0.96	0.96	**0.94**	0.94	0.9776~0.9802
InceptionV3	0.89	**0.97**	**0.97**	**0.93**	0.93	0.9790~0.9841
DenseNet201	0.90	0.97	0.97	0.93	0.93	**0.9812~0.9860**
InceptionResNetv2	0.88	0.97	0.97	0.92	0.93	0.9758~0.9813
EfficientNetb0	0.83	0.95	0.94	0.88	0.89	0.9520~0.9598

**Table 8:** Performance of the deep learning models with final fully connected layer replaced. The bold indicates the best.

Models	Sensitivity	Specificity	Precision	*F*1_*score*_	Accuracy	AUC(95% confidence interval)
Vgg19	**0.91**	**0.95**	**0.95**	**0.93**	**0.93**	**0.9757~0.9811**
ResNet50	0.90	0.94	0.94	0.92	0.92	0.9736~0.9793
InceptionV3	0.87	0.97	0.97	0.91	0.92	0.9736~0.9792
DenseNet201	0.89	0.97	0.96	0.92	0.93	0.9783~0.9834
InceptionResNetv2	0.80	0.94	0.93	0.86	0.87	0.9398~0.9486
EfficientNetb0	0.75	0.91	0.90	0.82	0.83	0.9061~0.9172

**Table 9:** Detection performance with varied α with *Width*,*Rate* fixed at 299 and 0.3, respectively.

*α*	Sensitivity(%)	*p* – *value_Sen_*	FPI	*p* – *value_FPI_*
0.5	82.20	0.70	1.44	0.97
0.6	**83.33**	-	1.44	-
0.7	80.51	0.34	1.42	0.84
0.8	81.64	0.57	**1.39**	0.57
0.9	77.12	0.04	1.52	0.37
1.0	77.97	0.08	1.51	0.42
1.1	78.25	0.09	1.46	0.84

**Table 10:** Detection performance with varied *Width* when *α* and *Rate* is 0.6 and 0.3.

*Width*	Sensitivity(%)	*p* – *value_Sen_*	FPI	*p* – *value_FPI_*
129	70.06	≪ 0.05	4.77	≪ 0.05
169	75.71	0.01	3.72	≪ 0.05
199	77.68	0.06	2.94	≪ 0.05
224	77.68	0.07	2.43	≪ 0.05
256	81.36	0.50	1.91	≪ 0.05
299	**83.33**	-	**1.44**	-

**Table 11:** Detection performance of the proposed framework on CBIS-DDSM dataset when α=0.6 and *Width* = 299.

*Rate*	Sensitivity(%)	*p* – *value_Sen_*	FPI	*p* – *value_FPI_*
0.2	79.10	0.005	**1.21**	≪ 0.05
0.3	83.33	0.16	1.44	≪ 0.05
0.4	83.90	0.23	1.70	≪ 0.05
0.5	85.31	0.47	2.07	≪ 0.05
0.6	86.44	0.76	2.44	0.003
0.7	**87.29**	-	2.86	1
0.8	**87.29**	1	3.21	0.03

**Table 12:** Detection performance of the proposed framework on INbreast dataset when α=0.6, where A and B in A@B stand for sensitivity and FPI, respectively. For p-value calculation, two way is carried out

	*Width*			
*Rate*	224	256	299	*P_S_* @ *P_F_*
0.2	87.16@0.98	87.16@0.66	88.99@0.44	
0.3	90.83@1.21	88.99@0.81	89.91@0.55	
0.4	92.66@1.36	89.91@0.93	91.74@0.64	≪ 0.05@
0.5	**95.41@1.69**	91.74@1.14	91.74@0.78	≪ 0.05
0.6	95.41@1.93	91.74@1.29	92.66@0.92	
0.7	95.41@2.27	92.66@1.51	92.66@1.05	
*P_S_* @ *P_F_*		0.007@≪ 0.05		

**Table 13:** Detection performance of the proposed framework on INbreast dataset when α=0.7, where A and B in A@B stand for sensitivity and FPI, respectively.

	*Width*			
*Rate*	224	256	299	*P_S_* @ *P_F_*
0.2	82.57@0.98	84.40@0.66	87.16@0.44	
0.3	86.24@1.21	87.16@0.81	88.07@0.55	
0.4	89.91@1.36	88.99@0.93	90.83@0.64	≪ 0.05@
0.5	92.66@1.69	94.50@1.14	91.74@0.78	≪ 0.05
0.6	92.66@1.93	**96.33@1.29**	93.58@0.92	
0.7	94.50@2.27	96.33@1.51	92.66@1.05	
*P_S_* @ *P_F_*		0.28@≪ 0.05		

**Table 14:** Detection performance of the proposed framework on INbreast dataset when α=0.8.

	*Width*			
*Rate*	224	256	299	*P_S_* @ *P_F_*
0.2	85.32@0.92	84.40@0.62	88.99@0.35	
0.3	88.07@1.10	86.24@0.78	91.74@0.46	
0.4	91.74@1.31	89.91@0.94	91.74@0.56	≪ 0.05@
0.5	**94.50@1.53**	89.91@1.13	91.74@0.69	≪ 0.05
0.6	94.50@1.79	90.83@1.32	93.58@0.79	
0.7	94.50@2.14	92.67@1.59	93.58@1.01	
*P_S_* @ *P_F_*		0.01@≪ 0.05		

**Table 15:** Method comparison.

Method	Dataset	Number of images for evaluation	Sensitivity @ FPI
Andreadis et al. (2020)	CBIS-DDSM	73	0.81@1.62
Cha et al. (2019)	CBIS-DDSM	361	0.83@0.04
Bandeira Diniz et al. (2018)	DDSM	~ 54	0.90@0.88
Divyashree and Hemantha Kumar (2021)	CBIS-DDSM	200	**0.94@–**
Lbachir et al. (2021)	CBIS-DDSM	152	0.91@0.65
Our method	CBIS-DDSM	**348**	0.87@2.86
Hassan Shayma’a et al. (2019)	INbreast	75	0.94@0.67
Kozegar et al. (2013)	INbreast	107	0.87@3.67
Shen et al. (2020)	INbreast	32	0.88@0.50
NiroomandFam et al. (2021)	INbreast	82	0.98@1.43
Cao et al. (2021)	INbreast	107	0.93@0.5
Agarwal et al. (2019)	INbreast	**410**	0.95@0.79
Our method	INbreast	107	**0.96@1.29**
